# Microbial Community in High Arsenic Shallow Groundwater Aquifers in Hetao Basin of Inner Mongolia, China

**DOI:** 10.1371/journal.pone.0125844

**Published:** 2015-05-13

**Authors:** Ping Li, Yanhong Wang, Xinyue Dai, Rui Zhang, Zhou Jiang, Dawei Jiang, Shang Wang, Hongchen Jiang, Yanxin Wang, Hailiang Dong

**Affiliations:** 1 State Key Laboratory of Biogeology and Environmental Geology, China University of Geosciences, Wuhan, 430074, PRC; 2 School of Environmental Studies, China University of Geosciences, Wuhan, 430074, PRC; 3 Institute of Earth Sciences, China University of Geosciences, Beijing, 10083, China; 4 Department of Geology and Environmental Earth Science, Miami University, Oxford, OH, 45056, United States of America; Wageningen University, NETHERLANDS

## Abstract

A survey was carried out on the microbial community of 20 groundwater samples (4 low and 16 high arsenic groundwater) and 19 sediments from three boreholes (two high arsenic and one low arsenic boreholes) in a high arsenic groundwater system located in Hetao Basin, Inner Mongolia, using the 454 pyrosequencing approach. A total of 233,704 sequence reads were obtained and classified into 12–267 operational taxonomic units (OTUs). Groundwater and sediment samples were divided into low and high arsenic groups based on measured geochemical parameters and microbial communities, by hierarchical clustering and principal coordinates analysis. Richness and diversity of the microbial communities in high arsenic sediments are higher than those in high arsenic groundwater. Microbial community structure was significantly different either between low and high arsenic samples or between groundwater and sediments. *Acinetobacter*, *Pseudomonas*, *Psychrobacter* and *Alishewanella* were the top four genera in high arsenic groundwater, while *Thiobacillus*, *Pseudomonas*, *Hydrogenophaga*, *Enterobacteriaceae*, *Sulfuricurvum* and *Arthrobacter* dominated high arsenic sediments. Archaeal sequences in high arsenic groundwater were mostly related to methanogens. Biota-environment matching and co-inertia analyses showed that arsenic, total organic carbon, SO_4_^2-^, SO_4_^2-^/total sulfur ratio, and Fe^2+^ were important environmental factors shaping the observed microbial communities. The results of this study expand our current understanding of microbial ecology in high arsenic groundwater aquifers and emphasize the potential importance of microbes in arsenic transformation in the Hetao Basin, Inner Mongolia.

## Introduction

Arsenic contamination in groundwater is a serious environmental issue in many countries such as Bangladesh, West Bengal, India, Burma, Vietnam, and China [1–2]. In China, populations at risk of exposure to excessive levels of arsenic (As) have been emerging since the 1960s [3]. Recent reports showed that about 19.6 million people are at risk of being affected by arsenic-contaminated groundwater [3–4]. Long time ingestion of arsenic groundwater can result in arsenicosis that causes many kinds of chronic diseases including cardiovascular, renal and respiratory diseases, as well as skin, lung, liver, kidney and prostate cancers [5–7].

Hetao Basin of Inner Mongolia is located in the arid-semiarid region in northwestern China and is one of the worst areas affected by arsenic poisoning in China [3, 4, 8, 9]. As concentrations in groundwater from this region are generally high, with some being more than 100 times the upper limit (10 μg/L) recommended by the World Health Organization [8, 10]. Local residents have been drinking high arsenic groundwater for over 20 years, resulting in more than 300, 000 cases of arsenicosis, seriously threatening public health and impacting sustainable development of the local economy [9].

Over the last decade, numerous hydrological, mineralogical and geochemical studies have been performed to investigate As mobilization and transformation mechanisms in the Hetao Basin [11–20]. There is a consensus that reductive dissolution of Fe oxide minerals and oxidation of pyrite release solid-phase As into groundwater. Previous studies have shown that As mobilization and transformation can be ascribed to complex interactions between microbes and geochemical processes [13, 21–22]. Recently, several studies have used traditional molecular methods such as denatured gradient gel electrophoresis (DGGE), terminal restriction fragment length polymorphism (TRFLP) and clone library analysis to characterize microbial communities in this basin [23–29]. Although these studies have yielded certain insights into the mechanisms of As mobilization and transformation, a comprehensive picture has not emerged, due to small numbers of samples studied and shallow sequencing depths used with these traditional methods [30]. Microbial communities in groundwater and sediments with contrasting As levels and geochemistry have yet to be fully understood. The important environmental factors shaping the microbial community structure are still poorly known. High-throughput sequencing approach, such as 454 pyrosequencing allows us to study a large set of samples across large geochemical gradients and at the same time to achieve a greater sequencing depth to capture rare microbes [31]. This characterization is important because arsenic-transforming microbes may be minor components in the overall community [32, 33]. Therefore, it is necessary for us to fill the above knowledge gap by using a high-throughput sequencing method coupled with rigorous statistical analysis.

Consequently, in this study, we used 454 pyrosequencing to: (1) reveal the diversity and structure of microbial communities in groundwater and sediments with different geochemistry; (2) assess the potential relationships between microbial communities and geochemical conditions, and subsequently (3) evaluate the putative roles of microorganisms in As release and mobilization in arsenic-rich aquifers of the Hetao Basin in Inner Mongolia of China. To achieve these objectives, a coordinated geochemical and molecular survey was conducted on 20 groundwater samples and 19 sediments from three boreholes in Hangjinhouqi County. The relationships among microbial diversity, community structure, and geochemistry were explored. The results of this study identify certain microorganisms that may be potentially important in regulating As biogeochemical transformation in the basin and expand our current understanding of As geomicrobiology in high As aquifers around the world.

## Materials and Methods

### Site description

No specific permission was required for the described field studies because no animal or human subjects were involved in this research. The sampling locations are not privately owned or protected in any way. The field studies did not involve endangered or protected species.

**Fig 1:**
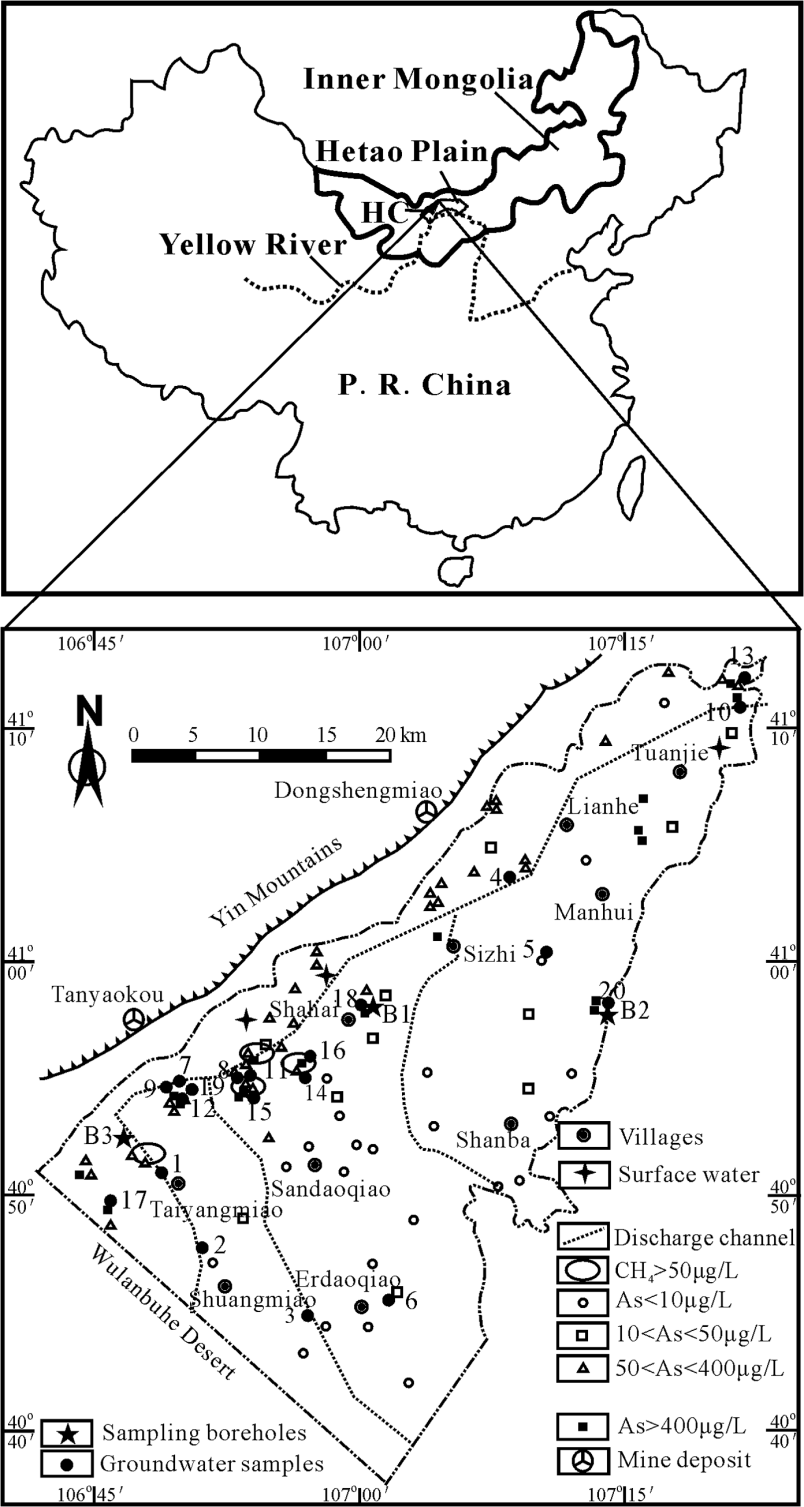
Map showing the location of the study area and the sampling sites. Numbers 1–20 refer to groundwater samples W1-20. Numbers B1-3 refer to three sampling boreholes.

The Hetao Plain is located in the western part of Inner Mongolia, China (Fig 1). The plain is formed at the end of Jurassic and contains fine clastic sediments. With the development of the Yellow River, frequent channels have deposited large amounts of sediments and generated oxbow lakes that have accumulated humus- and organic-rich mud. Shallow aquifers, the target of this study, are composed of late Pleistocene and Holocene alluvial and lacustrine deposits. The Hetao Plain has been one of the earliest irrigation districts using diverged Yellow River water. About half of the soils are saline, and soil salinization is further intensified by both strong evapotranspiration and seasonal irrigation. These semi-artesian aquifers are widely used for drinking by local residents in last several decades. Groundwater is recharged by vertical infiltration of meteoric water, laterally flowing groundwater from bedrocks, and/or by irrigation and leakage from the Yellow River on the south side. Discharge occurs mainly via evapotranspiration and pumping. Our case study was carried out in Hangjinhouqi County (HC) in the western part of the Hetao Plain where endemic arsenicosis is most serious [34]. Some of the local villagers were affected with serious skin lesions and cancers due to arsenicosis [35–36].

### Sample collection and field measurements

All the water samples were collected from drinking-water wells within a depth range of 20–30 m. High arsenic groundwater samples were collected in villages where arsenicosis is serious (Fig 1 and Table 1). Two boreholes (B1 and B2) were drilled at those sites where the groundwater shows the highest arsenic concentration and endemic arsenicosis is extremely serious. For comparison, low arsenic groundwater (W1-4) and sediment samples (B3.1-B3.7 from borehole B3) were collected at the sites with no arsenicosis (Fig 1). In total, twenty groundwater samples and nineteen sediment samples were collected for this study (Fig 1). Of the groundwater samples, three were collected in Taiyangmiao (sample W1, 2 and 17), 2 in Erdaoqiao (sample W3 and 6), 1 in Sizhi (sample W5), 1 in Manhui (sample W4), 10 located in Shahai (sample W7, 8, 9, 11, 12, 14, 15, 16, 18 and 19), 2 in Tuanjie (sampleW10 and 13), and 1 in Shanba (sample W20). Nineteen sediment samples were collected from three boreholes, and of which 7 samples from high As borehole B1 named as B1.1-B1.7(107°00'33.8''E, 40°58'01.0''N), 5 samples from high As borehole B2 named as B2.1-B2.5(107°14′02.5″E, 40°58′44.8″N), and 7 samples from low As borehole B3 named as B3.1-B3.7 (106°53 ′50.9″E, 40°55′3.2″N) (Fig 1). Sediments were collected from the three boreholes along different depths (Table 2). The sediments from the three boreholes ranged in texture from clay, silt to fine-medium sand and in color from brown, green, dark grey to totally black (Table 2).

**Table 1:** Geochemical parameters and major ion concentrations of groundwater samples.

Sample No.	T-As (μg/L)	As(Ⅲ) (μg/L)	ORP (mV)	TOC (mg/L)	NH_4_^+^ (mg/L)	D-S (μg/L)	Fe (μg/L)	Cl (mg/L)	Fe(Ⅱ)/ Fe(Ⅲ)	NO_3_^-^ (μg/L)
W1	2	1	3	3.8	0.7	BD	233	244.5	0.3	ND
W2	2	1	194	2.4	0.4	40	114	991.6	0.23	ND
W3	3	2	143	2.3	0.7	2	95	794.9	0.2	ND
W4	9	6	119	3.9	1.9	187	BD	1503.8	ND	0.9
W5	66	5	213	2.7	1.1	BD	1060.0	1492.3	0.34	244.0
W6	82	15	150	1.9	3.2	7	113.0	271.0	0.25	60.0
W7	304	238	-46	5	1.4	4	224	402.4	0.68	2.1
W8	359	267	-18	6.9	6.8	165	610.0	558.6	0.68	3.3
W9	401	285	-68	6.7	6.4	182	918.0	328.2	0.31	0.2
W10	416	220	26	6.9	3.2	23	510.0	380.4	0.81	4.8
W11	436	347	-65	3.8	5.3	140	200.0	514.9	0.31	2.6
W12	640	245	0	6.1	3.4	68	2258.0	402.2	1.24	11.0
W13	666	500	-166	9	6.7	131	805.0	337.5	0.49	1.9
W14	744	478	-190	8.9	4.0	15	480	80.3	0.85	33
W15	866	763	-69	18.4	6.4	BD	500	740.0	0.9	2.5
W16	1001	367	-47	8	5.2	31	557.5	589.4	0.44	9.0
W17	917	452	-22	10.7	5.4	29	400.0	72.7	2.56	9.0
W18	936	449	-74	11.7	7.2	BD	607.5	206.2	3.7	16.0
W19	1022	558	-16	6.9	6.8	14	324.0	684.4	1.84	2.2
W20	1088	582	-278	9.3	5.4	28	874.0	575.4	5.2	6.1

T-As: Total arsenic

D-S: Dissolved sulfide

BD: below detection limit

ND: not determined

**Table 2:** Geochemical parameters and major ion concentrations of sediments from two high arsenic boreholes and one control borehole.

Sample No.	Lithology	Depth(m)	As (mg/kg)	Fe (g/kg)	SO_4_^2-^ (mg/kg)	Total S (mg/kg)	TOC (mg/kg)	As(Ⅲ)/ As(Ⅴ)	Fe(Ⅱ)/ Fe(Ⅲ)
B1.1	□ I	1.0	77.7	3.62	0.223	2.3	5.63	0.02	0.01
B1.2	△ II	11.0	33.1	6.28	0.032	0.4	2.25	0.05	1.50
B1.3	◇ II	15.0	42.1	7.11	0.116	1.0	2.76	0.33	0.79
B1.4	△ IV	20.0	33.5	5.48	0.044	0.5	0.22	0.47	2.10
B1.5	△ IV	24.0	37.1	7.67	0.301	0.4	0.16	0.29	0.53
B1.6	△ II	29.0	34.4	5.17	0.29	0.5	0.19	0.30	12.61
B1.7	△ IV	32.6	32.6	6.56	0.089	6.1	0.02	0.52	2.15
B2.1	○ I	7.0	47.6	6.52	0.011	23.5	18.54	0.05	0.23
B2.2	○I IV	8.5	51.4	1.51	0.008	16.9	0.59	0.06	0.99
B2.3	◇II III	18.9	38.7	4.95	0.011	8.7	0.16	0.16	4.38
B2.4	◇II III	28.0	55.5	5.31	0.089	6.1	0.16	0.29	3.02
B2.5	◇II III	30.0	48.4	8.08	0.013	11.0	0.19	0.29	1.01
B3.1	○ II III	4.3	2.9	0.82	0.182	0.5	9.81	ND	40.00
B3.2	□ I	7.0	4.1	0.87	0.201	0.9	11.6	ND	4.80
B3.3	□ I	10.5	5.8	1.78	0.493	1.2	11.16	ND	7.48
B3.4	◇III IV	16.6	5.2	5.4	0.899	2.0	13.69	ND	1.70
B3.5	△ IV	22.1	1.9	4.4	0.449	0.6	2.83	ND	2.41
B3.6	○IV	24.7	1.7	2.37	0.501	0.5	2.08	ND	2.54
B3.7	△ IV	30.0	1.5	0.51	0.042	0.1	2.5	ND	4.10

I: brown, II: green; III: dark grey, IV: black

□ clay, ○ silty clay, ◇ medium sand, △ fine sand

ND: not determined

Water samples were pumped, and filtered. The tubing was flushed substantially prior to each use. Samples used for total soluble As, Fe, NH_4_^+^ and dissolved total sulfide were filtered through 0.45 μm mixed cellulose ester membrane filters. The filtrates were acidified to pH<2. To determine total dissolved sulfide, water samples were collected into acid-washed polyethylene bottles, preserved with zinc acetate and sodium hydroxide on-site to stabilize the sulfide. Arsenic speciation separation was accomplished with disposable syringes and silica-based strong anion exchange cartridges (Supelco, USA) at each sampling point following a previous method [27]. Microbial samples were collected by filtration of 5–10 L water through 0.2-μm filters (Millipore), and biomass-containing filters were wrapped and placed in a 50 mL sterile conical centrifuge tube. For sediment sampling, boreholes were drilled using a 12-cm diameter piston-coring device to depth of 30 to 33 m, and sediment samples were immediately packed into polyethylene bags and sealed. All groundwater and sediment samples were immediately frozen and stored on dry ice in the field and during transportation (within two days), and then kept at -80°C in laboratory until further analysis.

### Geochemistry measurements

Anion and As concentrations were measured according to previously published methods [27, 37]. Briefly, arsenic concentration in water samples was determined by using liquid chromatography-hydride generation-atomic fluorescence spectrometry (LC-HG-AFS, Haiguang AFS-9780, Beijing). Concentrations of anions, including SO_4_^2-^, NO_3_^-^, Cl^-^, and F^-^ were determined by ion chromatography (DX-120, Dionex, USA). NH_4_^+^ was determined by the Nessler’s reagent colorimetric method [8]. Total dissolved sulfide concentration was determined from precipitated zinc sulfide using the methylene blue method [8]. Total and ferrous iron concentrations were determined with the 1,10-phenanthroline-based assay [38]. Methane in water samples was analyzed with MAT-271 mass spectrometer at the Lanzhou Institute of Geology, Chinese Academy of Sciences. Total organic carbon (TOC) was measured using a TOC analyzer (TOC-VCPH, Shimadzu Corporation, Japan).

As speciation, SO_4_^2-^ and total sulfur (T-S), total and ferrous iron concentrations, and TOC content in sediment samples were measured according to previously published methods [39–41]. Before extraction, external layers of sediment cores were removed and each sediment sample was homogenized in an anaerobic chamber filled with 95% N_2_ and 5% H_2_ (Coy Laboratory Products, Grass Lake, MI, USA). Arsenic was extracted from 0.2 g of each sediment sample with orthophosphoric acid and ascorbic acid. The mixture was maintained in microwave (MARSXpress, CEM Corp., USA) at 60 W for 10 min [40, 41]. For total sulfur concentration measurement, sediment samples of 1 gram in weight were digested with a microwave digestion technique in a HNO_3_-HF-H_3_BO_3_ solution and heated at 100–110°C for 40 min. Sulfate was extracted using 0.01 M Ca(H_2_PO_4_)_2_ (pH = 4.0) as extractant [42]. Total and ferrous iron was extracted from 0.1 g sediment samples with 1.8 M H_2_SO_4_ and 48% HF and determined with the 1,10-phenanthroline assay [43]. TOC contents of sediment samples were measured using a TOC analyzer (Vario MICRO cube, Elementar, Hanau, Germany). All samples were run in triplicate and then averaged.

### DNA extraction and amplification of 16S rRNA genes

Before DNA extraction, external layers of sediment cores were removed to avoid possible contamination. DNA extractions for all groundwater and sediments samples were conducted using the FastDNA spin kit for soil (MP Bio, USA) according to manufacturer's manual. DNA yield was quantified with Nanodrop (Thermo Scientific NanoDrop 2000), and then stored at -80°C until PCR amplification. The bacterial and archaeal V4-V8 variable regions of the 16S rRNA gene were amplified with the modified forward primer 515F (5’-GTGCCAGCMGCCGCGGTAA-3’) in combination with the reverse primer 1391R (5’-GACGGGCGGTGWGTRCA-3’). Unique 8-bpbarcodes were added at the 5’-end of both the forward and reverse primers to identify the samples from the reads [44]. PCR was carried out under aseptic conditions using autoclaved and/or UV-treated plastic ware and pipettes. All PCRs were undertaken in Master cycler ep Gradient S Thermal Cycler block (Eppendorf, Hamburg, Germanny). A typical 50 μL PCR mixtures contained 0.4 pmol m/L of primers (MWG), 1 μL DNA template (20–50 ng), 10x reaction buffer (Promega Corporation), 5 mM MgCl_2_, 1.5 U Biotaq DNA polymerase (Promega), 0.25 m dNTP (Promega). PCRs were performed with the following thermo cycler program: denaturation at 95°C for 5 min, 30 cycles of denaturation at 94°C for 1 min, annealing at 55°C for 45 seconds and extension at 72°C for 1 min; and final extension at 72°C for 7 min [27].

### Pyrosequencing and data analysis

Pyrosequencing of the V4 region of the 16S rRNA gene was carried out from the 515F-end of the amplicons with the Roche (454) genome sequencer FLX+ system (454 Life Sciences, USA) at SeqWright Inc (Houston, USA). All data analyses were performed using the Quantitative insights into microbial ecology (QIIME) software package, version 1.5.0 (http://qiime.org/install/virtual_box.html#virtual-box) [45]. Low quality reads were filtered out using split_libraries.py, and then the remaining reads were denoised to reduce the amount of erroneous operational taxonomic units (OTUs). Chimeric sequences were identified with Chimera Slayer and excluded. The uclust method was used for OTU picking [46]. The most abundant sequence in each cluster was chosen as a representative. All representative sequences were aligned with Python nearest alignment space termination (PyNAST)[47]. Sequences were clustered against the Greengenes database (http://greengenes.lbl.Gov) at 97% identity using uclust [46]. The pyrosequencing reads were deposited at the European Molecular Biology Laboratory-European Bioinformatics Institute (EMBL-EBI) database under the accession number ERR699330.

### Statistical analysis of geochemical and microbial data

All microbial statistical analyses were performed in the QIIME pipe line and/or using the package “vegan” in R, version 3.1.0 (http://www.r-project.org/) [44–45, 48–49]. Chao1, Shannon, and equitability indices were calculated at the 97% cutoff level after removing samples with fewer than 1248 reads. These diversity indices were tested for their correlation with the geochemical data using Mantel test [44, 49]. Analysis of similarity (ANOSIM) in the community composition among certain pre-defined groups was performed based on Bray-Curtis dissimilarity at the 97% similarity level [44, 49]. The groups were pre-defined based on water and sediment geochemistry. Three comparisons were made among 4 pre-defined groups: 1) between 4 low and 16 high arsenic groundwater samples, 2) between 12 sediment samples from two high arsenic boreholes and 7 sediment samples from one control borehole, and 3) between 16 high arsenic groundwater and 12 high arsenic sediment samples. Similarity percentage (SIMPER) [50] analysis was performed to rank the top ten OTUs that contributed to the observed similarity (or dissimilarity) among the three comparisons described above. The average abundances of these OTUs in each group were then calculated. Jackknifed unweighted pair-group method with arithmetic means (UPGMA) clustering was used to compare the microbial community similarity among the samples based on Bray-Curtis dissimilarity at the 97% similarity OTU level. For principal coordinate analysis (PCoA), the Bray-Curtis dissimilarity among samples was constructed. PCoA and hierarchical clustering analyses were performed to identify important geochemical parameters that contributed to the observed difference of microbial community structure. Environmental variables were identified based on significance calculated from canonical correspondence analysis and variance inflation factors (VIFs) results before biota-environment matching analysis (BIO-ENV). The BIO-ENV procedure and co-inertia analysis (CIA, ad4 package) [51] were used to detect the relationships between microbial community composition and environment factors.

## Results

### Sample characteristics and geochemical composition

**Fig 2:**
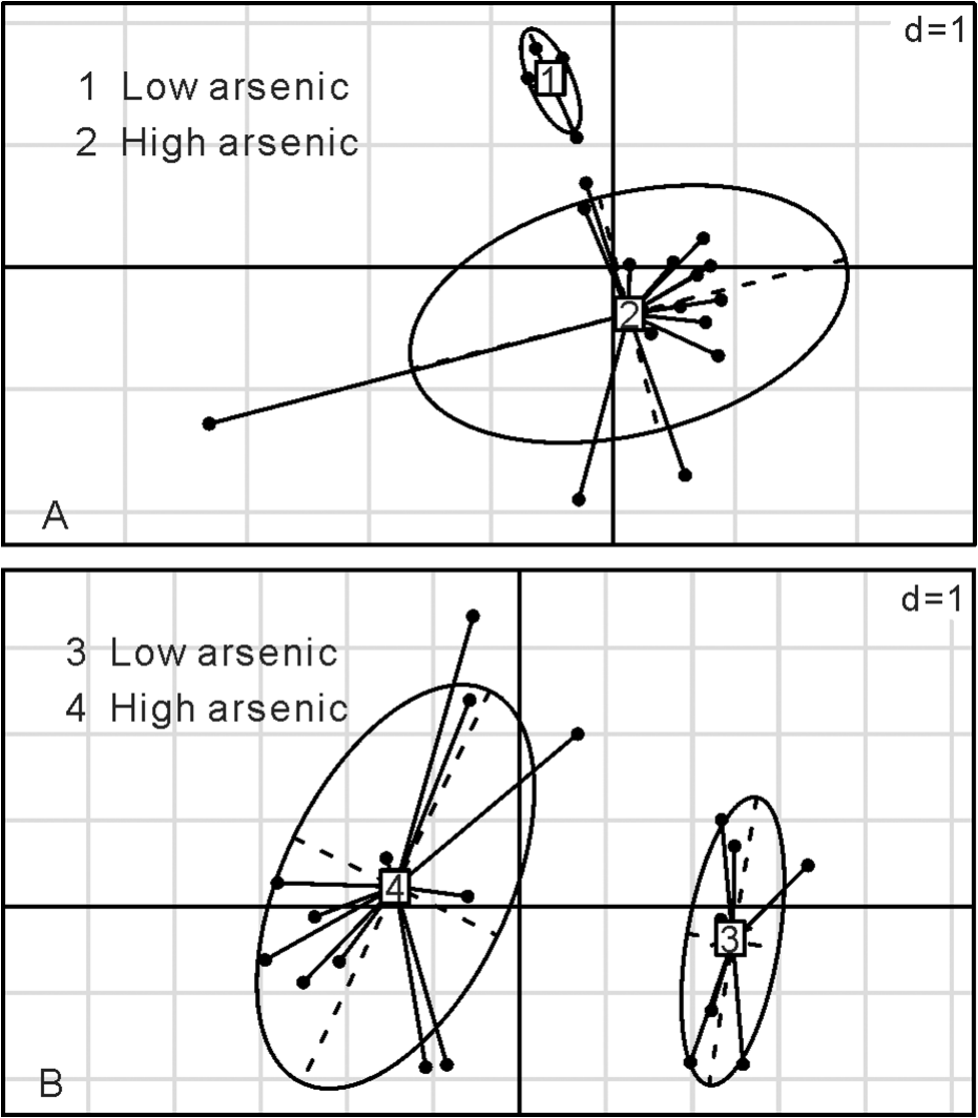
PCoA plots based on different arsenic concentrations. Each of the groups is numbered inside square boxes. The 20 groundwater and 19 sediment samples are indicated and linked to the boxes, respectively. The ellipses represent group clusters based on K-means clustering. The scale is given by the value in the upper right corner which represents the length of the side of background grid squares. A: groundwater samples; B: sediment samples. Group 1: arsenic concentrations between 2–9 μg/L in low arsenic groundwater; group 2: arsenic concentrations between 66–1088 μg/L in high arsenic groundwater; group 3: arsenic concentrations between 1.5–5.8 mg/kg in sediments from the control borehole B3; group 4: arsenic concentrations between 33.1–77.7 mg/kg in sediments from high arsenic borehole B1 and B2.

Ten environmental variables that were correlated with microbial communities of groundwater were selected on the basis of correlation identification (Table 1). PCoA and hierarchical clustering analyses divided the geochemistry of the 20 groundwater samples into two groups (Fig 2A and S1 Fig). The first group consisted of four samples (W1-4), which were characterized by a low arsenic content (<10 μg/L), relatively high oxidation reduction potential (ORP, mean value 115 mV), and low TOC and NH4^+^. The concentrations of CH_4_ in these samples were all below detection limit (1 ppmv). The second group included two sub-groups: an intermediate arsenic concentration group (W7-13, 304–666 μg/L total As), and a high arsenic concentration group (W14-20, 744–1088 μg/L As). Most samples in the second group were either neutral or slightly alkaline in pH. The ORP of these samples were negative with the medium and mean values of -47.0 mV and -51.65 mV, respectively. Some samples in this group had high sulfide concentrations. Arsenic concentrations were generally high in the samples that had low concentrations of sulfate, negative ORP, high concentrations of As(III), high ratios of Fe(II) to Fe(III), and high TOC content. Such geochemical characteristics indicated that a strong reducing condition prevailed in the high arsenic groundwater samples.

Eight environmental variables were identified to be correlated with microbial communities in the sediments (Table 2). PCoA (Fig 2B) and hierarchical clustering (S1 and S2 Figs) analyses geochemically divided all the sediment samples into two well-defined groups: a high arsenic group (including samples B1.1-B1.7 and B2.1-B2.5) and a control group (samples B3.1-B3.7 from the control borehole). The total arsenic (T-As) concentrations in the sediments from the two high arsenic boreholes ranged from 33.1 to 77.7 mg/kg. The As(III) concentrations and As(III)/total As ratios increased with depth. In contrast, As(V) generally decreased with depth. Total Fe concentrations and the ratios of Fe(II) to Fe(III) ranged from 1.51–8.08 g/kg and 0.01 to 12.61, respectively, and showed an overall increasing trend with depth in high arsenic samples. SO_4_^2-^ concentrations and total sulfur varied between 8–301 mg/kg and 0.4–23.5 g/kg, respectively. For the sediments from control borehole B3, total arsenic concentrations were distinctly lower than those in the two high As boreholes B1 and B2, with a range from 1.5 to 5.8 g/kg. Total sulfur and total Fe (T-Fe) concentrations in these sediments from the control borehole were relatively lower with mean values of 2.31 g/kg and 0.83 mg/kg, respectively, but the ratios of sulfate to total sulfur (mean value 0.52) were distinctly higher than those in high arsenic sediments (mean value 0.14).

### Alpha diversity of microbial community

A total of 233,704 reads were obtained for the 39 groundwater and sediment samples after removal of low-quality and chimeric sequences. A variety of taxa were observed at the 97% OTU level, with 12–267 observed and 23–355 predicted OTUs (based on Chao1). Coverage values ranged from 31.3% to 81.6% (Table 3). For the groundwater samples, the average species richness (Chao1), Shannon diversity, and equitability for the high arsenic group were lower than those for the low arsenic group. Mantel tests failed to identify correlations between these diversity indices and T-As, As(III), and any other measured geochemical variables in the groundwater samples. However, the diversity indices for the sediment communities were moderately correlated with SO_4_^2-^ concentration and the ratio of SO_4_^2-^ to T-S (SO_4_^2-^ and Simpson: r = 0.44, p = 0.03; SO_4_^2-^ and equitability: r = 0.35, p = 0.007; SO_4_^2-^ /T-S and Shannon: r = 0.24, p = 0.047; SO_4_^2-^ /T-S and Simpson: r = 0.50, p = 0.001).

**Table 3:** Alpha diversity indices at the 97% OTU level of groundwater and sediment samples with sequence number higher than 1248.

Sample No.	Chao1	Observed OTU	Coverage* of the observed OTUs (%)	Shannon	Simpson	Equitability
W1	227	71	31.28	3.36	0.79	0.55
W2	138	85	61.63	2.55	0.52	0.40
W3	299	214	71.57	5.78	0.93	0.75
W4	355	267	74.65	6.58	0.97	0.86
W5	43	33	76.55	2.35	0.72	0.47
W6	66	26	39.39	1.12	0.32	0.24
W7	349	242	69.32	6.36	0.96	0.80
W8	89	69	77.53	3.80	0.87	0.62
W9	76	44	58.18	1.92	0.51	0.35
W10	113	74	65.27	2.83	0.65	0.46
W11	35	26	74.29	1.54	0.42	0.33
W12	137	89	64.85	3.16	0.67	0.49
W13	45	29	65.02	1.12	0.27	0.23
W14	46	31	67.39	1.02	0.24	0.21
W15	103	76	73.65	3.43	0.75	0.55
W16	60	41	68.19	2.52	0.65	0.47
W17	267	136	51.02	3.42	0.65	0.48
W18	163	129	79.05	4.09	0.80	0.58
W19	96	38	39.69	1.24	0.28	0.24
W20	80	49	61.54	2.22	0.58	0.39
B1.1	293	231	78.81	7.03	0.99	0.89
B1.2	163	130	79.75	5.30	0.95	0.75
B1.3	347	195	56.25	6.13	0.96	0.81
B1.4	170	100	58.90	3.58	0.77	0.54
B1.5	98	69	70.23	4.11	0.88	0.67
B1.6	67	53	79.10	2.83	0.71	0.49
B1.7	139	101	72.49	3.62	0.79	0.54
B2.1	152	84	55.14	3.98	0.89	0.62
B2.2	176	99	56.25	2.92	0.70	0.44
B2.3	73	50	68.40	2.09	0.52	0.37
B2.4	62	44	71.54	2.32	0.65	0.42
B2.5	144	105	72.89	4.44	0.88	0.66
B3.1	120	72	59.77	3.29	0.78	0.53
B3.2	140	105	74.76	4.11	0.81	0.61
B3.3	117	64	54.79	2.44	0.55	0.41
B3.4	91	74	81.56	2.13	0.43	0.34
B3.5	72	51	70.61	1.21	0.25	0.21
B3.6	23	12	53.33	0.40	0.09	0.11
B3.7	87	48	55.49	3.69	0.88	0.66

*Coverage is the ratio of the observed OTUs to Chao1.

### Microbial community composition

The top ten taxonomic groups that contributed to the dissimilarities or similarities among the three defined groups were identified by the SIMPER analysis (Table 4). Microbial community structure was markedly different between the groundwater and sediment samples of high arsenic concentration. Although the top ten bacterial taxa at the family/genus level were present in both sample groups (Table 4, first panel), their relative abundances were very different. *Acinetobacter*, *Psychrobacter* and *Alishewanella* were more abundant in the groundwater than in the sediments. The average abundances of *Thiobacillus*, *Pseudomonas*, *Hydrogenophaga*, *Enterobacteriaceae*, *Sulfuricurvum* and *Arthrobacter* were higher in the sediments than in the groundwater samples. *Acinetobacter* and *Thiobacillus* contributed the most to the dissimilarities between the high arsenic groundwater and sediments (average abundance 30.45% vs. 12.31%, respectively, see Table 4). *Acinetobacter* was the predominant genus in the groundwater (62.41%), whereas *Thiobacillus* was the most abundant (average abundance 24.62%) in the sediments but absent in most groundwater samples (average abundance 0.01%).

**Table 4:** Top ten taxonomic groups identified by SIMPER (at the 97% level) that contributed to dissimilarity among different sample groups.

**High arsenic groundwater (a) VS sediment samples (b)**				
Taxon	Family/Genus	Contribution^1^ (%)	Avg^2^ ^.a^ (%)	Avg^2^ ^.b^ (%)
***Proteobacteria***	*Acinetobacter*	30.45	62.41	1.82
***Proteobacteria***	*Thiobacillus*	12.31	0.01	24.62
***Proteobacteria***	*Pseudomonas*	5.23	7.90	11.88
***Proteobacteria***	*Psychrobacter*	3.21	5.79	1.09
***Proteobacteria***	*Alishewanella*	3.00	5.95	0.08
***Proteobacteria***	*Hydrogenophaga*	2.82	0.05	5.67
***Proteobacteria***	*Enterobacteriaceae*	2.48	0.01	4.96
***Proteobacteria***	*Sulfuricurvum*	2.35	0.01	4.69
***Actinobacteria***	*Arthrobacter*	2.34	0.25	4.58
***Bacteroidetes***	*Flavobacterium*	1.71	1.72	2.21
**High (a) VS low (b) arsenic groundwater samples**				
Taxon	Family/Genus	Contribution^1^ (%)	Avg^2^ ^.a^ (%)	Avg^2^ ^.b^ (%)
***Proteobacteria***	*Acinetobacter*	31.06	62.41	0.29
***Proteobacteria***	*Pseudomonas*	12.18	7.90	28.45
***Bacteroidetes***	*Gillisia*	5.24	0.00	10.48
***Proteobacteria***	*Psychrobacter*	3.31	5.79	1.40
***Proteobacteria***	*Aeromonas*	3.19	0.00	6.37
***Proteobacteria***	*Alishewanella*	3.03	5.95	0.18
***Firmicutes***	*Natronobacillus*	2.75	0.20	5.54
***OP3***	*Unclassified OP3*	2.56	0.56	5.33
***Firmicutes***	*Exiguobacterium*	2.53	0.53	4.90
***Firmicutes***	*Sporosarcina*	2.00	0.53	3.00
**Sediments from the high arsenic boreholes (a) VS the control borehole (b)**				
**Taxon**	Family/Genus	Contribution^1^ (%)	Avg^2^ ^.a^ (%)	Avg^2^ ^.b^ (%)
***Proteobacteria***	*Enterobacteriaceae*	14.82	4.96	30.01
***Proteobacteria***	*Psychrobacter*	13.03	1.09	26.07
***Proteobacteria***	*Thiobacillus*	12.31	24.62	0.00
***Proteobacteria***	*Pseudomonas*	10.70	11.88	16.76
***Actinobacteria***	*Arthrobacter*	2.84	4.58	1.81
***Proteobacteria***	*Hydrogenophaga*	2.84	5.67	0.00
***Firmicutes***	*Sporosarcina*	2.61	1.80	4.76
***Proteobacteria***	*Sulfurimonas*	2.35	4.69	0.00
***Proteobacteria***	*Massilia*	1.76	0.08	3.49
***Bacteroidetes***	*Flavobacterium*	1.10	2.21	0.00

^1^ Contribution of each OTU to the overall dissimilarity between these two clusters (a and b).

^2^ Average abundance of each OTU in these two clusters (a and b).

There were large differences in the microbial community structures between the low and high arsenic samples. The predominant groups in high arsenic groundwater were *Acinetobacter*, *Psychrobacter* and *Alishewanella*. Two genera including *Gillisia* and *Aeromonas* were only present in low arsenic groundwater. Other genera including *Natronobacillus*, *Unclassified OP3*, *Exiguobacterium*, and *Sporosarcina* were much more abundant in low arsenic groundwater than in high arsenic groundwater. For the sediments, *Thiobacillus*, *Hydrogenophaga*, *Sulfurimonas* and *Flavobacterium* were present only in the high arsenic sediments, but *Enterobacteriaceae*, *Psychrobacter*, *Pseudomonas*, *Sporosarcina* and *Massilia* were much more predominant in the control sediments. Some microbial groups were highly abundant in certain samples. For example, *Hydrogenophaga* was predominant in samples D1.5 and D1.7. The percentages of *Arthrobacter* in sample B2.1 and B2.2 were significantly higher than in other sediment samples.

The archaeal abundances in high arsenic groundwater and sediment samples were mostly lower than 1%. Methanogens were the predominant archaea in most high arsenic groundwater. Five groundwater samples (W7, 12, 15, 17 and 18) contained > 24.4% methanogens out of total archaea.

### Overall microbial community structure in relation to geochemistry

**Fig 3:**
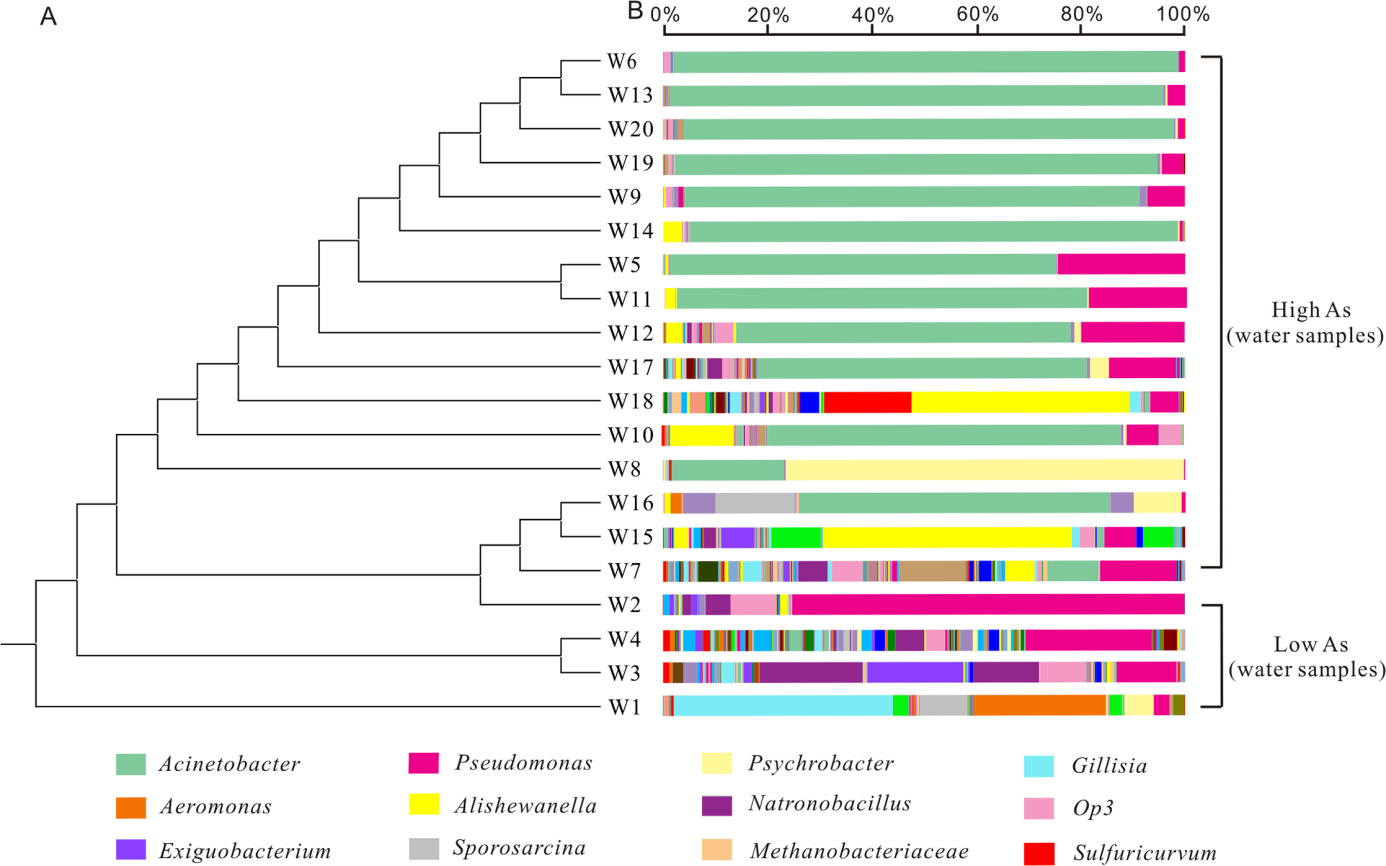
Microbial community compositions of groundwater samples grouped by arsenic concentrations. A. UPGMA cluster tree based on Bray-Curtis dissimilarity at the 97% similarity OTU level; B. microbial compositions at the genus or family level. The legend showed the top twelve OTUs (at the 97% level).

**Fig 4:**
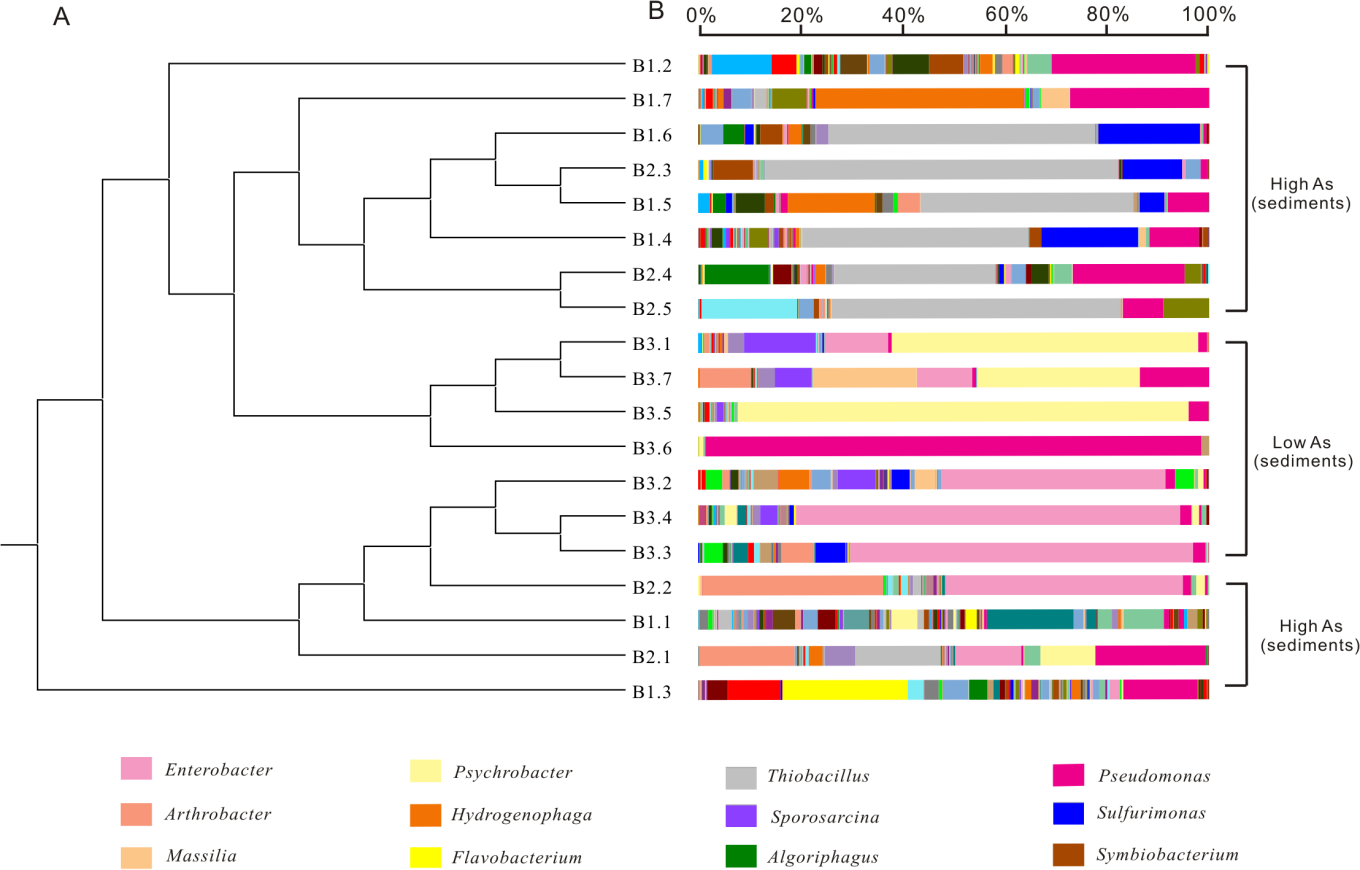
Microbial community compositions of sediment samples grouped by arsenic concentrations. A: UPGMA cluster tree based on Bray-Curtis dissimilarity at the 97% similarity OTU level; B: microbial compositions at the genus or family level. The legend showed the top twelve OTUs (at the 97% level).

**Fig 5:**
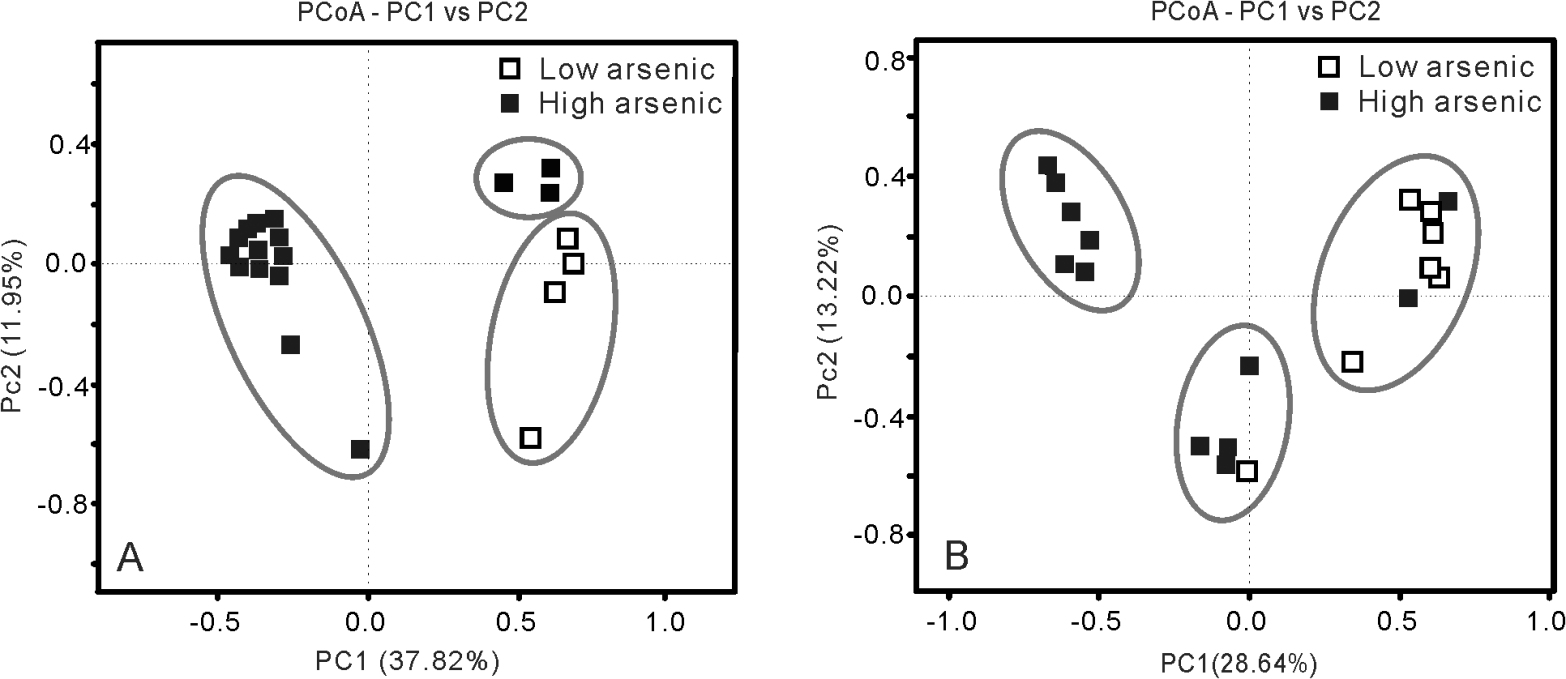
PCoA plots based on microbial communities of 20 groundwater (A) and 19 sediment samples (B). The first two factors P1 and P2 for the groundwater samples can explain 37.82% and 11.95% variations. P1 and P2 for the sediment samples can explain 28.64% and 13.62% variations. Solid and hollow squares indicate high and low As groundwater/sediment samples, respectively.

The results of pairwise comparisons using ANOSIM suggested that there were significant differences in the microbial community structure among the identified four groups: 1) between the low and high arsenic groundwater samples; 2) between the high arsenic and low arsenic sediments; 3) between the high arsenic groundwater samples and sediments, with the R-statistic values of 0.709, 0.429 and 0.64, respectively and all p values < 0.01 in these three comparisons. Similar result was also identified based on Bray-Curtis dissimilarity at the 97% similarity OTU level using UPGMA or hierarchical cluster trees (Figs 3, 4 and S3 Fig). For example, the low and high arsenic groundwater samples formed two distinct groups. For the sediments, the grouping pattern identified by UPGMA was also similar to that by ANOSIM (Fig 4). PCoA plots of beta diversity results showed that the microbial communities of both groundwater samples and sediments were divided into two distinctly different groups based on As concentrations (Fig 5).

**Fig 6:**
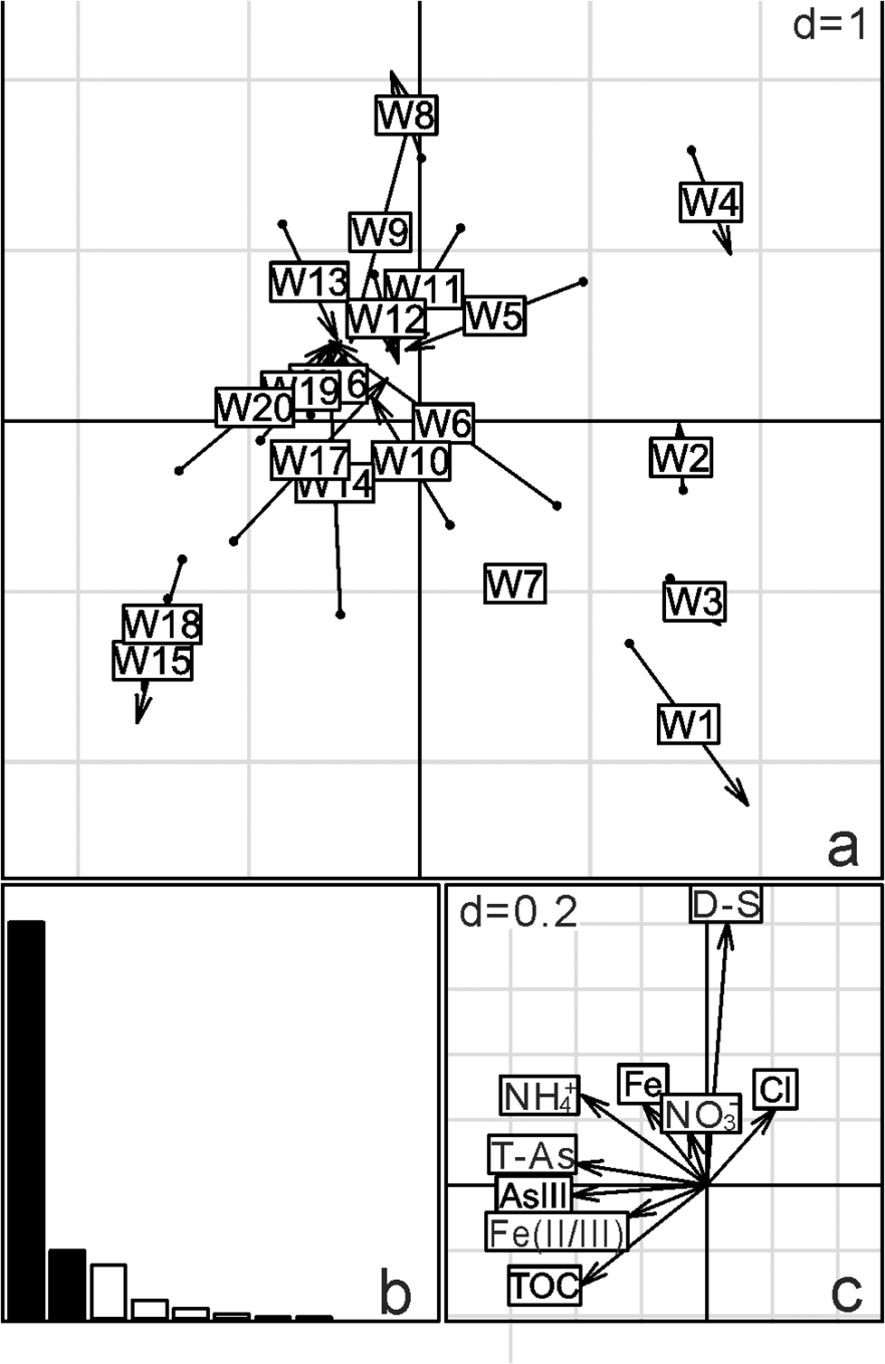
Co-inertia (CIA) analysis of the relationships between microbial community composition and environment factors for 20 groundwater samples. The scale is given by the value in the upper right corner which represents the length of the side of background grid squares. (a) Samples projected on the first two axes of the analysis. These axes account for 68% and 16% of the variation, respectively. (b) Histogram of the eigenvalues corresponding to the two co-inertia axes, which are equal to 2.15 and 0.35. (c) Main geochemical vectors that affect sample ordination in the CIA. The lengths of the vector arrows represent the influence of the given geochemical parameters on the co-structure of the CIA. Main geochemical vectors are represented by their chemical symbols, total arsenic is given as T-As and dissolved sulfide is given as D-S. p-value = 0.07.

**Fig 7:**
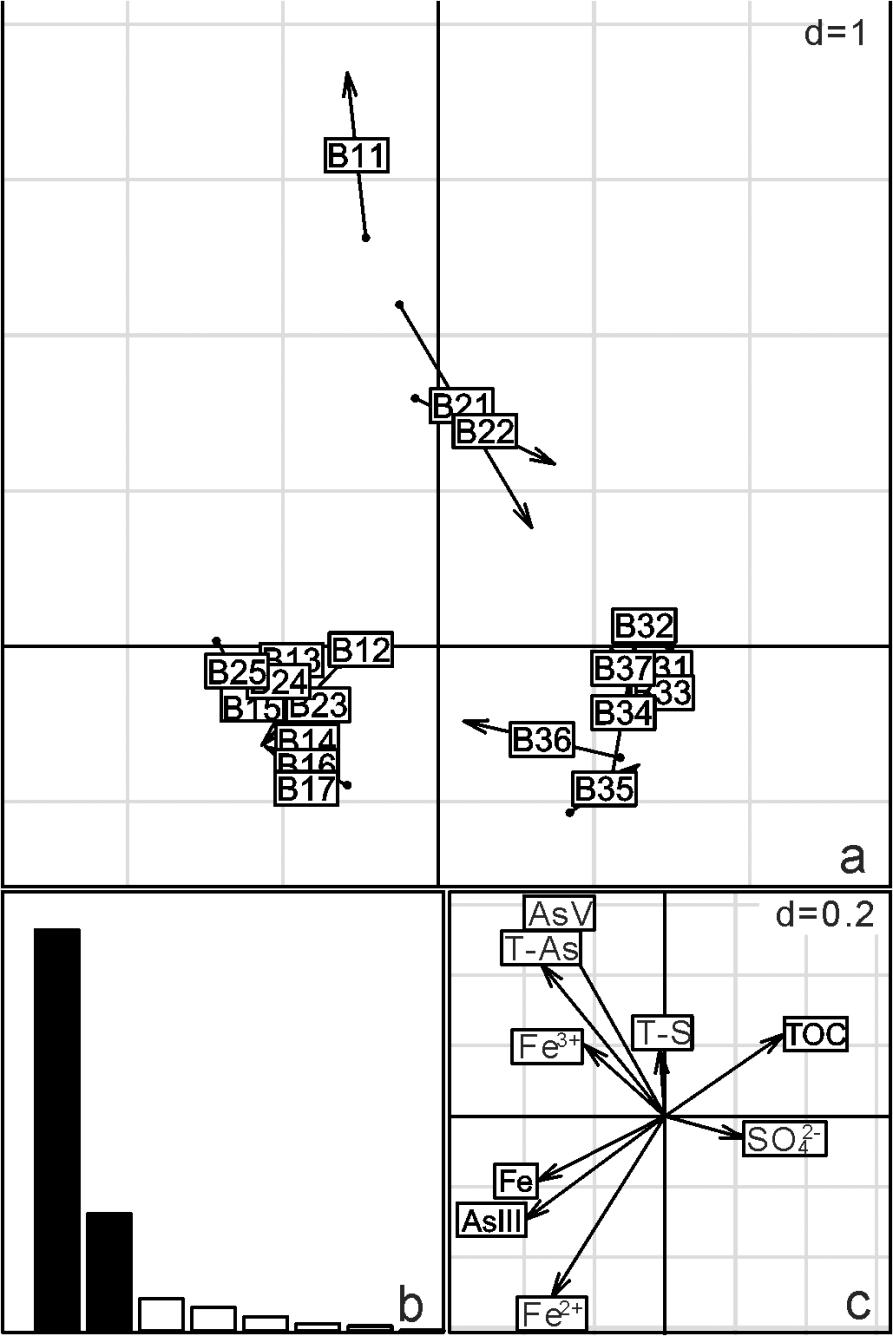
Co-inertia analysis (CIA) of the relationships between 19 sediment samples microbial community composition and environment factors. The scale is given by the value in the upper right corner which represents the length of the side of background grid squares. (a) Samples projected on the first two axes of the analysis. These axes account for 55% and 26% of the variation, respectively. (b) Histogram of the eigenvalues corresponding to the two co-inertia axes, which are equal to 3.15 and 0.85. (c) Main geochemical vectors that affect sample ordination in the co-inertia. The lengths of the vector arrows represent the influence of the given geochemical parameters on the co-structure of the CIA. Main geochemical vectors are represented by their chemical symbols, total arsenic is given as T-As and total sulfur is given as T-S. p-value = 0.002.

The BIO-ENV results confirmed that the community structure of the groundwater samples was correlated with concentrations of total As, As(III), TOC and NH_4_^+^ (r = 0.36). For the sediments, the community structure was moderately correlated with a combination of T-As, As(III)/As(V), SO_4_^2-^/T-S and Fe^2+^ concentration (r = 0.44). In order to visualize the relative importance of geochemical vectors that affected sample ordination, co-inertia analysis was carried out between microbial community and geochemistry (Figs 6 and 7). The length and direction of a geochemical vector represent the degree of influence of that vector on the microbial community structure. The first two axes of the co-inertia analysis explained 83% and 80% of the total environmental variability in the groundwater and sediment samples, respectively. Co-inertia analysis showed the relationships between microbial community composition and environment factors were marginally significant and significant for 20 groundwater and 19 sediment samples, respectively (groundwater: p = 0.07, r = 0.351; p = 0.002, r = 0.507). Similar to the results of BIO-ENV, CIA showed that microbial communities in high arsenic groundwater were associated with T-As, As(III), TOC and NH_4_^+^, and microbial communities in high arsenic sediments were associated with T-As, As(III), T-Fe and Fe^2+^.

## Discussion

### Important environmental factors shaping the microbial community structure

Arsenic is one of the key environmental factors that contribute to the difference in the geochemistry and microbial community structure in both groundwater and sediments. Groundwater and sediment samples could be divided into well-defined high and low arsenic groups based on both geochemistry and microbial community structure. Previous research has shown that high arsenic aquifers were characterized by high concentrations of total dissolved Fe, NH_4_^+^ and S^2−^, high TOC content, low concentrations of NO_3_^−^ and SO_4_^2−^, which are different from low arsenic aquifers [9, 6]. Our results are consistent with these previous studies. This geochemical difference could be explained by the mechanisms of arsenic release and mobilization. Reductive dissolution of As bearing Fe-oxides, hydroxides, and oxyhydroxides and sulfate may release Fe(II), As(V) and sulfide into aqueous solution, which may result in the geochemical characteristics of high arsenic aquifers [12, 16, 21]. High arsenic in groundwater and sediments may challenge certain microbial populations [23, 24] and favor As-resistant ones. This might be the reason that microbial populations in high arsenic samples were distinctly different from those in low arsenic samples.

The dominant microbial community populations were also distinctly different between the high arsenic groundwater and sediment samples (Table 4). Such difference in dominant microbial community might be related to difference in the geochemical conditions. Most of the high arsenic groundwater samples were under a strong reducing condition as evidenced by negative ORP, high concentrations of Fe(II), As(III), H_2_S, CH_4_, and low concentrations of SO_4_^2-^. In contrast, high arsenic sediments were under a weakly reducing condition, as evidenced by low ratios of As(III)/As(V) and high ratios of SO_4_^2-^/total sulfur in most samples. The contrasting redox condition between groundwater and sediment, even at the same depth, may be due to redox disequilibrium between them. It is faster for flowing groundwater to each redox equilibrium than low-permeability sediment rich in clays and silt (Table 2). These differences in the redox condition and geochemistry were mostly consistent with the observed differences in the dominant microbial community populations between high arsenic groundwater and sediment samples. For examples, *Acinetobacter*, *Psychrobacter* and *Alishewanella* that were previously identified to be capable of reduction [25, 52–54] were predominantly found in our strongly reducing groundwater samples. Oxidizing bacteria *Thiobacillus*, *Hydrogenophaga* and *Sulfuricurvum* [55–57] dominated the sediment samples.

### Predominant populations in high arsenic aquifers in Hetao Basin

The predominant groups detected using the 454 pyrosequencing approach in this study were consistent with previous results derived from traditional sequencing methods [27–29]. However, the relative abundances of the dominant populations were different. The bacterial communities identified in this study were more abundant and diverse than those detected in previous studies using traditional sequencing methods. For example, *Alishewanella*, *Sulfuricurvum*, *Arthrobacter*, *Sporosarcina* and *Algoriphagus* were not detected previously, but they were identified in the current pyrosequencing dataset. The identification of these genera is likely due to the greater sequence depth of the pyrosequencing method, but also the effects of primer sets and PCR bias can not be ruled out either [58].

A few previous studies have investigated microbial communities in high arsenic aquifers, mostly in Bangladesh [23, 24], Datong Basin in China [25], Red River Delta, Vietnam, and West Bengal [26, 27] with traditional culture-dependent and culture-independent methods such as DGGE, TRFLP and clone library analysis. To our best knowledge, this is the first study to investigate microbial community in high arsenic aquifers across several important geochemical gradients with high throughput sequencing approach. In high arsenic groundwater samples, the dominant groups in our study area were different from those in high arsenic tube wells in Bangladesh (such as *Acidovorax*, *Acinetobacter*, *Hydrogenophaga* [23], *Pseudomonas*, *Elizabethkingia* and *Pantoea* [24]). These differences may be related to different geochemical conditions such as ORP [24]. In contrast, high arsenic sediment communities in our study site, *Thiobacillus*, *Hydrogenophaga* and *Arthrobacter* are similar to those in high arsenic sediments from Red River Delta and West Bengal [26, 59]. Likewise, some genera such as *Pseudomonas*, *Acinetobacter* and *Arthrobacter* have been found in the high arsenic aquifers in Shanyin County, Datong Basin, China [25].

### Potential roles of microorganisms in As release and mobilization

Based on sequence similarity to cultured representatives, we speculate that abundant processes in our high arsenic samples appeared to be related to arsenic resistance, arsenate reduction, sulfur oxidation, hydrogen oxidation, and denitrification. For example, *Acinetobacter* sp. strain GW7, *Alishewanella* sp. strain GIDC-5, *Pseudomonas mendocina* strain GW9 and *Pseudomonas putida* strain WB have been found to be involved in As cycling including arsenic resistance, arsenic reduction and oxidation [25, 52, 60]. In addition to *Pseudomonas* and *Arthrobacter*, some oxidizing bacteria such as *Thiobacillus*, *Hydrogenophaga*, and *Sulfuricurvum* also dominated our high arsenic sediments. Some *Thiobacillus* spp. and *Sulfuricurvum* spp. were identified to be capable of oxidizing iron, sulfur and thiosulfate [55, 61, 62]. *Hydrogenophaga* spp. was reported as a facultative autotroph capable of oxidizing hydrogen and arsenite and growing on different inorganic electron donors such as arsenite, ammonium, nitrite, and several sulfur species [56, 57].

In our previous study, a large amount of pyrite (FeS_2_) containing high concentrations of structural As was found in the sampling site [39]. Pyrite might provide rich metabolic substrates (Fe and S) to the predominant population *Thiobacillus*, one kind of putative iron and sulfur-oxidizing bacterium, resulting in As(V) accumulation onto oxidized mineral surfaces. At greater depth when geochemical condition became more reducing, As(V) could be transformed into As(III) through microbial reduction (by *Psychrobacter*, *Pseudomonas*, and *Arthrobacter*). The resulting As(III) could then be released into groundwater due to its much higher mobility than As(V) [63] and move around along with flowing groundwater both laterally and vertically. Such a mechanism would explain why As(V) was dominant in most high As sediment samples, while As(III) was the prevalent As species in groundwater [64].

## Supporting Information

S1 Fig Hierarchical clustering analysis based on geochemical parameters of groundwater samples. Samples were divided into two well-defined groups with high and low arsenic concentrations.
(TIF)

S2 Fig Hierarchical clustering analysis based on geochemical parameters of sediment samples from two high arsenic boreholes and one control borehole. Samples were divided into two well-defined groups with high and low arsenic concentrations.
(TIF)

S3 Fig Hierarchical clustering analysis based on Bray-Curtis dissimilarity of high arsenic sediment and groundwater microbial communities. Most of the high arsenic groundwater and sediment samples were divided into two well-defined groups.
(TIF)
